# Interaction of milrinone with extracorporeal life support

**DOI:** 10.1051/ject/2024014

**Published:** 2024-12-20

**Authors:** Aviva J. Whelan, Sabiha Mim, J. Porter Hunt, Autumn M. McKnite, Danielle J. Green, Carina E. Imburgia, Jeremiah D. Momper, Gideon Stitt, Kevin M. Watt

**Affiliations:** 1 Division of Clinical Pharmacology, Department of Pediatrics, University of Utah 295 Chipeta Way Salt Lake City UT 84108 USA; 2 Division of Pediatric Critical Care, Department of Pediatrics, University of Utah 295 Chipeta Way Salt Lake City UT 84108 USA; 3 Pharmacometric Research Group, Department of Pharmacy, Uppsala University Husargatan Uppsala 752 37 Sweden; 4 Department of Pharmacology and Toxicology, University of Utah 295 Chipeta Way Salt Lake City UT 84108 USA; 5 Skaggs School of Pharmacy and Pharmaceutical Sciences, University of California San Diego 9255 Pharmacy Lane, MC 0657 La Jolla CA 92093 USA

**Keywords:** Milrinone, Extracorporeal life support (ECLS), Extracorporeal membrane oxygenation (ECMO), Continuous renal replacement therapy (CRRT)

## Abstract

*Background:* Milrinone is commonly prescribed to critically ill patients who need extracorporeal life support such as extracorporeal membrane oxygenation (ECMO) and continuous renal replacement therapy (CRRT). Currently, the effect of ECMO and CRRT on the disposition of milrinone is unknown. *Methods*: *Ex vivo* ECMO and CRRT circuits were primed with human blood and then dosed with milrinone to study drug extraction by the circuits*.* Milrinone percent recovery over time was calculated to determine circuit component interaction with milrinone. *Results:* Milrinone did not exhibit measurable interactions with the ECMO circuit, however, CRRT cleared 99% of milrinone from the experimental circuit within the first 2 hours. *Conclusion*: Milrinone dosing adjustments are likely required in patients who are supported with CRRT while dosing adjustments for ECMO based on these *ex-vivo* results are likely unnecessary. These results will help improve the safety and efficacy of milrinone in patients requiring ECMO and CRRT. Due to the limitations of *ex-vivo* experiments, future studies of milrinone exposure with ECLS should include patient circuit interactions as well as the physiology of critical illness.

## Introduction

Patients undergoing cardiopulmonary bypass (CPB) are at risk of low cardiac output syndrome (LCOS). LCOS and heart failure are conditions that frequently require support with extracorporeal life-support (ECLS) such as extracorporeal membrane oxygenation (ECMO) and continuous renal replacement therapy (CRRT) [1, 2]. Milrinone is frequently used in critically ill patients supported on ECLS and has been shown to reduce the risk of LCOS in children with heart failure or those who have undergone CPB [3–10].

ECLS is an important life-saving technology, however, patients supported with ECMO and/or CRRT are at high risk of mortality [1, 11–13]. The increased mortality risk may be in part due to altered drug disposition in patients on ECLS [14, 15]. ECLS can alter drug disposition in several ways: 1) adsorption of the drug to ECLS circuit components; 2) hemofilter clearance of the drug in CRRT; 3) exogenous fluids used in priming the circuit increase the volume of distribution [16]. Additionally, other factors such as edema, inflammation, and altered protein binding can affect drug disposition in the population of critically ill patients supported on ECLS. The degree of drug extraction by ECLS depends on the circuit components as well as drug physicochemical properties (e.g., protein binding, molecular weight, lipophilicity). Hydrophilic drugs with low protein binding are likely to be cleared by a hemofilter, while lipophilic and highly protein-bound drugs more frequently adsorb to circuit components [17, 18].

Milrinone is a type 3 phosphodiesterase inhibitor that provides inotropic, lusitropic, and vasodilatory effects, making it an important agent in treating patients with heart failure and LCOS. It increases cyclic adenosine monophosphate levels which in turn increases intracellular calcium concentrations. Calcium can then be used by troponin I and phospholamban which impact inotropy and lusitropy, respectively [19–21]. In adults, plasma milrinone concentrations ranging from 100 to 300 ng/mL are associated with therapeutic effects [22, 23]. Toxicities like systemic hypotension from excessive vasodilation, arrhythmias, and thrombocytopenias are associated with higher concentrations (>500 ng/mL) [23, 24]. Since little is known about milrinone disposition in ECLS, patients are at risk for either treatment failure from subtherapeutic dosing or toxicity from excessive exposure. Therefore, there is an urgent need to understand how milrinone interacts with ECLS circuits.

The interactions between ECLS circuits and individual drugs have been studied in *ex vivo* experiments where a blood-primed ECLS circuit is dosed with a drug and concentrations are measured over time [16, 25–29]. The extent of drug adsorption to the circuit components, impact of surface coatings, and drug clearance by the hemofilter can be assessed by this method. We performed *ex vivo* CRRT and ECMO experiments in this study to quantify the interaction of milrinone with ECLS to determine the degree of extraction and inform optimal dosing. Since milrinone is mildly protein-bound, we hypothesize that it will likely be readily filtered by CRRT and adsorb minimally to ECMO circuits.

## Materials and methods

We evaluated the extraction of milrinone from blood-primed ECMO and CRRT circuits. ECMO and CRRT circuits were each set up in a closed loop and primed with a blood solution using previously published methods [16, 30, 31]. Circuits were then dosed with milrinone to achieve therapeutic concentrations (200 ng/mL) [22, 23], and concentrations were measured over time to quantify the extent of milrinone extraction by the circuit. A control sample containing the same prime solution was also dosed to achieve similar concentrations (200 ng/mL) that were measured over time to understand milrinone degradation [19, 20].

### Extracorporeal membrane oxygenation (ECMO) setup

**Figure 1:**
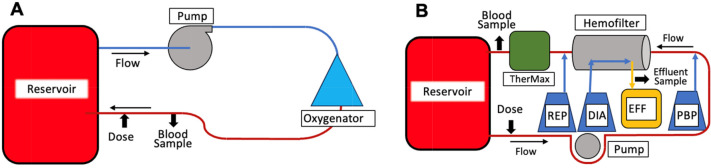
Schematic of ECMO and CRRT *ex vivo* circuit configurations: A. ECMO circuit configuration including oxygenator, reservoir, and pump. B. CRRT circuit configuration including reservoir and PrisMax (pump, hemofilter, thermax and fluids). PBP = pre-blood pump, EFF = effluent, DIA = dialysate, REP = replacement fluid.

ECMO *ex-vivo* experiments were conducted as previously reported [31]. In short, ECMO circuits (*n* = 3) were set up as illustrated in Figure 1A and included: a reservoir (Viaflex 1000 mL, Baxter, Deerfield, IL); a centrifugal pump (Rotaflow Pump, Maquet); a hollow-fiber oxygenator (Quadrox-iD, Maquet, Hirrlingen, Germany); and tubing (Sorin Smart Perfusion Pack, LivaNova, London, UK) (Table 1). Circuits were primed with a mixture of Plasma-Lyte A crystalloid (~200–300 mL; Baxter Healthcare, Deerfield, IL), 1 unit of thawed human plasma frozen within 24 hours after phlebotomy (250-300 mL), 2 units of human red blood cells (adenine saline added leukocyte reduced [~600 mL]), tromethamine (2 g), heparin sulfate (500 units), sodium bicarbonate (7 mEq), calcium gluconate (650 mg), and human serum albumin (12.5 g) for a total priming fluid volume of about 1300 mL. To minimize the strain on hospital blood bank supply, expired blood products from the American Red Cross were used to prime the circuits. In order to maintain the physiologic pH (7.2–7.5), CO_2_ and tromethamine were added to the priming solution and as necessary during the experiment. ECMO circuit flow was maintained at 1.0 L/min (HT110 with H8XL flow sensor, Transonic, Davis, CA). A Quadrox-iD integrated heat exchanger was connected to an ECMO water heater (Cincinnati Sub-Zero, Cincinnati, OH) to maintain the temperature of the priming solution at 37 °C. The haemoglobin of this solution was 10 g/dL.

**Table 1:** ECMO and CRRT circuit components and parameters.

ECMO milrinone circuits	Oxygenator	Pump	Tubing^c^	Hemofilter	Reservoir	# of Doses
Run 1–3	Quadrox iD Adult^a^ Bioline^b^	Rotaflow Bioline^b^	Sorin Smart-X^d^	–	Viaflex	1

CRRT milrinone circuits	System		Hemofilter		Temp Control	Reservoir
	Baxter PrisMax		HF1000^e^		TherMax bag^f^	EXACTAMIX EVA^g^
	PBP (mL/h)	BFR (mL/min)	PFR (mL/h)		DIA (mL/h)	REP (mL/h)
Run 1–3	700	150	0		1000	300

^a^Polymethylpentene fibers; ^b^Bioline coating: covalently bonded recombinant human albumin and heparin; ^c^Polyvinyl chloride; ^d^Smart-X coating: tribloc copolymer (polycaprolactone-polydimethylsiloxane-polycaprolactone) ^e^PolyarylEtherSulfone fibres, plasticized polyvinyl chloride tubing; ^f^Ethylene vinyl acetate; ^g^Polyurethane. PBP: pre-blood pump flow rate; BFR: blood flow rate; PFR: patient fluid removal flow rate; DIA: dialysate volumetric flow rate; REP: replacement fluid flow rate.

### Continuous renal replacement therapy (CRRT) setup

Three CRRT *ex-vivo* experiments were run using the PrisMax system (Baxter Healthcare, Deerfield, IL) with HF1000 filters as previously reported [31]. In short, the HF1000 filter set was set up in a closed loop by connecting it to a reservoir (EXACTAMIX EVA, Baxter Healthcare). The system was primed with 0.4 units of fresh frozen plasma (125 mL), 1 unit of packed red blood cells (300 mL), Plasma-Lyte A crystalloid (150 mL), tromethamine (1.5 g), heparin sulfate (350 units), sodium bicarbonate (7 mEq), calcium gluconate (180 mg), and human serum albumin (6.25 g) for a total priming fluid volume of ~ 600 mL. To maintain physiologic pH additional tromethamine was added as needed. The blood mixture temperature was kept at 37 °C by a TherMax blood warmer. At our institution, continuous venovenous hemodiafiltration (CVVHDF) is the modality used for critically ill patients, thus this was the mode selected for these experiments. The following prescription was used: blood flow rate (BFR) 9000 mL/h, dialysis solution flow rate (DIA) 1000 mL/h, pre-blood pump fluid (PBP) 700 mL/h, total replacement solution flow rate (REP) 300 mL/h delivered after filtration, and patient fluid removal flow rate net 0 mL/h (Table 1), for a total effluent rate of 2000 mL/h. PrismaSATE 4/2.5 Dialysis Solution (Baxter Healthcare, Deerfield, IL) was used for dialysis, a pre-blood pump, and all replacement fluids.

### Control setup

To determine drug degradation in the blood mixture throughout the experiment, three control experiments were included. For each control, 50 mL of blood priming solution was drawn from the ECMO circuits before drug dosing and placed into polypropylene centrifuge tubes (229426, CELLTREAT, Pepperell, MA). These samples were dosed to achieve similar concentrations as the circuits and incubated in a water bath heated to 37 °C for the duration of the experiment.

### Drug administration and sample collection

Milrinone was administered to the ECMO circuits via a three-way stopcock downstream to the sampling port on the arterial limb (Figure 1A). One dose of milrinone was administered to each circuit at time zero and blood samples were collected at 1, 5, 15, 30, 60, 120, 180, 240, and 360 min after drug administration.

Milrinone was administered to CRRT circuits via an access line downstream of the reservoir (Figure 1B), blood was sampled from the post-filter sampling port, and effluent was sampled from the post-filter effluent sampling port. Milrinone was administered at time zero and blood and effluent samples were collected at 1, 5, 15, 30, 60, 120, 180, 240, and 360 min. Blood samples were centrifuged at 3000*g* and 4 °C for 10 minutes immediately after collection. Plasma and effluent samples were then frozen in cryovials (Fisher Scientific, Pittsburgh, PA) at −20 °C for < 6 h and stored at −80 °C until analysis.

Control samples were dosed with milrinone at time = −5 min, gently rotated to ensure adequate mixing, and then placed in a water bath for the duration of the experiment. At each sample time point, the tube was removed from the water bath, gently inverted five times, and the sample was collected.

### Milrinone assay

Calibrators and quality control samples were prepared using standard reference materials. Milrinone and the internal standard (milrinone-d3) were obtained from Cayman Chemical (Cat# 13357 and 25429, respectively). Calibration standards were prepared in Mass Spect Gold Human Plasma (Cat#: MSG7000; Golden West Diagnostics) and were used to generate an external 7-point calibration curve (2, 6, 13.5, 30, 75, 300, and 600 ng/mL) using linear regression (1/*x* weighting) to plot the peak area ratio versus concentration. The calibration curves were linear (*R*^2^ ≥ 0.99) over the analytically measurable range (AMR) of 2–600ng/mL. Within-run precision of quality control samples (QCs, *n* = 3) was determined at 3 different concentrations in plasma (Low QC: 4.5 ng/mL, Mid QC: 45 ng/mL, High QC: 450 ng/mL) were observed at 6.3%, 14.2%, and 7.3%, respectively. Quantitative determination of milrinone in plasma and effluent samples was determined by multiple reaction monitoring (MRM) LC-MS/MS (Agilent Infinity II 1290 – Sciex QTrap 6500+). MRM was performed in positive electrospray ionization mode by monitoring the following transition ions: milrinone (quantifier: 212.05 m/z > 142.0 m/z; qualifier: 212.05 m/z > 104.0 m/z; milrinone-d3 (IS): 215.07 m/z > 142.1 m/z).

### Milrinone recovery over time

Due to slight differences in initial concentrations for circuit and control experiments, concentrations were normalized using drug percent recovery using the following equation:

(1) $$\mathrm{Recovery}\left(\;\%\right)=\frac{C_{t}}{C_{\mathrm{ref}}}∙100$$

where *C*_t_ is the concentration at time *t* and *C*_ref_ is the initial concentration of milrinone at time 1 min.

### Saturation coefficient

The saturation coefficient and transmembrane clearance were calculated for the CRRT experiments using paired plasma and effluent samples with the following equations:

(2) $$S_{a(\mathrm{HDF})}=\;\frac{C_{\mathrm{eff}}}{C_{p}}$$

(3) $$Q_{\mathrm{eff}}=Q_{\mathrm{PBP}}+Q_{\mathrm{REP}}+Q_{\mathrm{PFR}}+Q_{\mathrm{DIA}}$$

(4) $${\mathrm{CL}}_{\mathrm{CVVHDF}}=Q_{\mathrm{eff}}∙S_{a(\mathrm{HDF})}$$

where *S*_a(HDF)_ is the saturation coefficient for hemodiafiltration and *C*_eff_ and *C*_P_ are the effluent and plasma milrinone concentrations, respectively. *Q*_eff_, *Q*_PBP_, *Q*_REP_, *Q*_PFR_ and *Q*_DIA_ are the effluent, pre-blood pump, replacement fluid, patient fluid removal, and dialysate volumetric flow rates, respectively. CL_CVVHDF_ is the transmembrane clearance.

### Milrinone circuit clearance

Milrinone *ex vivo* clearance from the CRRT circuit was calculated using the equation:

(5) $$\mathrm{CL}=\;\frac{\mathrm{Dose}}{\mathrm{AUC}}$$

where the dose is 0.1 mg, the amount of milrinone is given to the circuit, and AUC is the area under the curve for the measured milrinone concentrations over time in *ex vivo* CRRT circuits. AUC was calculated using R Version 4.2 using MESS and tidyverse.

### Statistics

We compared milrinone recovery between ECLS circuits and controls at time = 360 minutes using a two-sample *t*-test with significance defined as *p* < 0.05. The lower limit of quantitation (LLOQ) for this assay was 2 ng/mL. Thus, a value of 2 ng/mL was used in our calculations for samples that returned as <LLOQ.

## Results

### Milrinone in ECMO circuits

**Figure 2:**
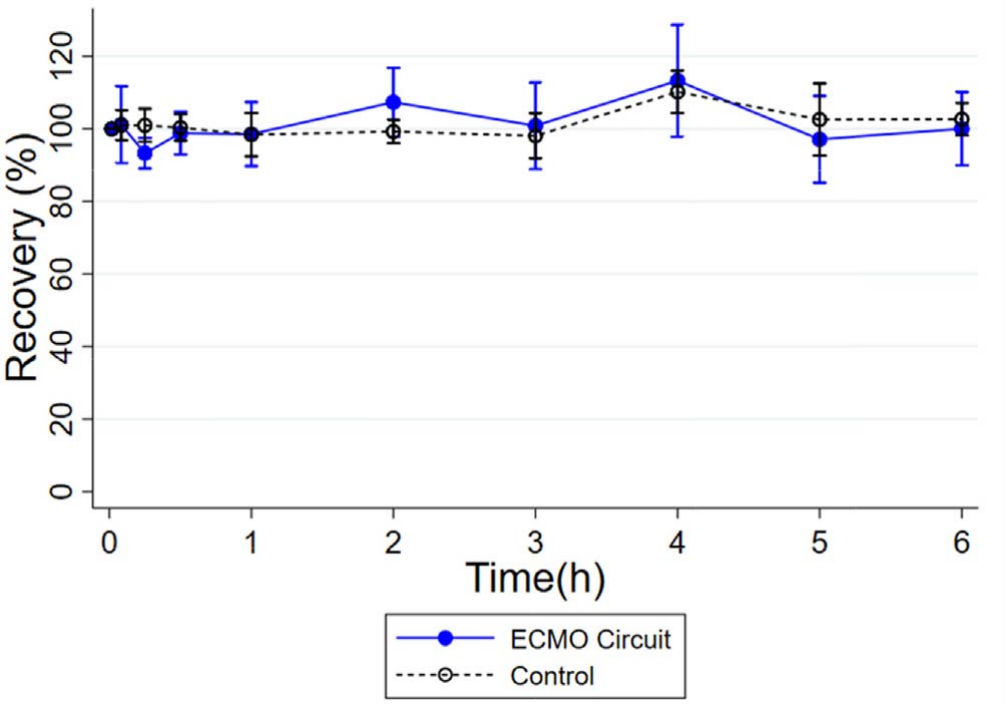
Plasma percent recovery from control and ECMO experiments for milrinone. Error bars represent one standard deviation for *n* = 3 for control and *n* = 3 for ECMO experiments.

Milrinone was minimally extracted by the ECMO circuit (Figure 2). The mean (standard deviation) recovery of milrinone in the ECMO circuit at *t* = 360 min was 100% (10.1). Recovery in the ECMO circuit was not significantly different compared to mean (standard deviation, SD) recovery in the control (103% [4.5]) at *t* = 360 min (*p* = 0.7). Control and ECMO milrinone plasma concentration measurements are reported in Supplementary Tables 1 and 2.

### Milrinone in CRRT circuits

**Figure 3:**
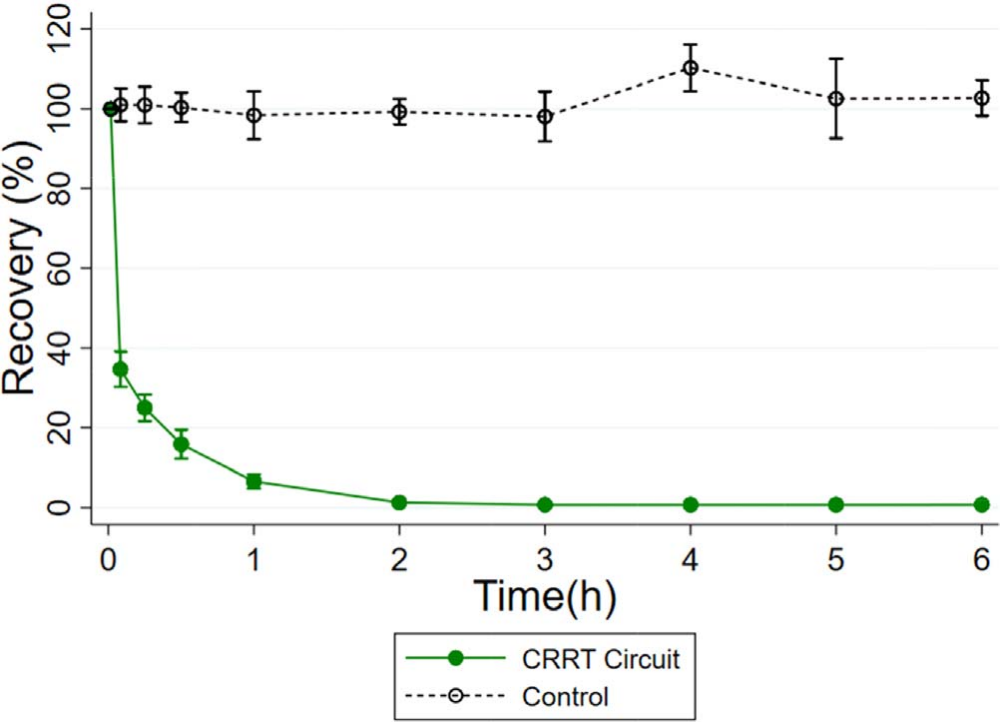
Plasma percent recovery from CRRT circuit experiments with milrinone. Error bars represent one standard deviation for *n* = 3 for control and *n* = 3 for CRRT (plasma).

Milrinone was rapidly extracted by the CRRT circuit (Figure 3) with milrinone concentrations below the lower limit of quantitation by 3 h. The mean (SD) recovery of milrinone in the CRRT circuit at *t* = 360 min was 0.73% (0.09). Recovery in the CRRT circuit was significantly different compared to mean (standard deviation) recovery in the control (103% [4.5]) at *t* = 360 min (*p* ≤ 0.001).

Because milrinone concentrations in plasma and hemofiltration were below the lower limit of quantitation after 2 h, the sieving coefficient was calculated using all time points up to 2 hours. The mean (SD) sieving coefficient across all CRRT circuits was 0.71 (0.23). This resulted in a mean (SD) milrinone transmembrane clearance of 119 mL/min (39.3). Control and CRRT milrinone plasma and effluent concentration measurements are reported in Supplementary Tables 1 and 3.

### Milrinone CRRT circuit clearance

Milrinone *ex vivo* AUC from minute 1 to hour 6, and clearance from the CRRT circuit by run is reported in Table 2 and Supplementary Table 2. The average clearance is 1350 mL/h (9.45 L/h/70 kg).

**Table 2:** Milrinone CRRT clearance effluent concentrations (ng/mL).

Time	CRRT circuit		
	Run 1	Run 2	Run 3
AUC_0.0167-6_	80.6	81.2	63.4
CL (mL/hr)	1240.6	1232.7	1578.0

## Discussion

In this study, we performed *ex vivo* ECLS experiments with milrinone to assess its interaction with ECMO and CRRT circuits. Milrinone recovery was not significantly different in ECMO experiments compared to the control (Figure 2) which suggests no adsorption to ECMO circuit components. Both the control and ECMO experiments showed steady concentrations of milrinone with no degradation over time. This contrasts with the CRRT experiments where milrinone was rapidly cleared.

To our knowledge, this is the first evaluation of milrinone interactions with ECMO circuits. Our ECMO experiments showed essentially no interaction of milrinone with the ECMO circuit with 100% recovery of milrinone over the 6-hour experiments. Prior studies with other drugs and ECMO showed adsorption to circuit components was more likely with highly lipophilic and protein-bound drugs [26, 32–37]. Milrinone is only slightly lipophilic (LogP 0.3–1) and moderately protein bound (70%) [19]. Therefore, these results are concordant with our hypothesis that the physicochemical properties of milrinone would result in minimal circuit-drug adsorption.

In contrast, milrinone was rapidly cleared by CRRT with concentrations below the lower limit of quantitation by 3 h. In a CRRT system, drugs can be extracted via adsorption to circuit components and/or clearance across the dialysis membrane. For milrinone, the sieving coefficient of 0.7 suggests that most of this loss was due to transmembrane clearance. While additional extraction via adsorption is possible, it is less likely given our findings in the ECMO system. Published pediatric PK literature reports milrinone clearance ranging from 2.91–17.6 L/h/70 kg [5, 38, 39]. The average clearance of milrinone calculated from these three *ex vivo* circuits was 9.45 L/h/70 kg and falls within the lower end of the reported range.

These results provide additional insight regarding two published studies describing milrinone exposure in a small cohort of adults (*n* = 6) [40] and children (*n* = 3) [38] supported with CRRT. Both studies reported that milrinone clearance was lower in individuals on CRRT compared to those with normal renal function, this is expected as milrinone is renally cleared and total clearance depends on both clearance by CRRT and native renal function. Additionally, clearance due to continuous venovenous hemofiltration estimated in the adult study was lower than the clearance determined through this work. This suggests that the CVVHDF modality results in greater drug clearance than the CVVH modality. These observations confirm that native renal function and CRRT modality, as well as filter type and dialysis prescription, should be incorporated when dosing milrinone in patients on CRRT [29, 30].

There are limitations to this work. Milrinone is frequently used as an infusion in patients in the intensive care unit (ICU) and this study only included a single bolus dose. Infusion dosing can have an impact on ECLS by saturating adsorption sites. Given that minimal adsorption was observed, this is unlikely to have a substantial impact on our results. Additionally, these experiments were carried out with only one type of ECMO and CRRT circuit components, hemofilters, and surface coatings making it difficult to generalize to circuits using different materials. Another limitation of this study is that milrinone CRRT circuits were all run at the same flow rates that are comparable to flow rates used clinically but do not encompass the heterogeneity seen in clinical practice. Based on our results, flow rates that reflect more aggressive dialysis will increase drug clearance by CRRT [31]. Finally, optimal milrinone dosing on ECLS cannot be confirmed using *ex vivo* experiments in isolation as these studies fail to account for patient factors such as organ function, edema, variations in plasma proteins, and patient-circuit factors like increased volume of distribution.

Nevertheless, these results demonstrate that optimal dosing of milrinone in patients on CRRT must account for clearance by the CRRT circuit as well as residual renal function. Future studies that consider important patient pathophysiology are needed to better predict milrinone exposure. We have developed an approach that uses physiologically based pharmacokinetic (PBPK) modelling to translate results from ex vivo experiments into optimal dosing recommendations [41]. PBPK models are structured in a physiologically relevant manner with virtual organ compartments connected by blood flow. Each virtual “organ” is parameterized with mass-balance differential equations characterizing the disposition of the drug within the compartment. In order to model drug exposure in patients on ECLS, an ECLS “organ” can be linked to the PBPK model and parameterized using data from *ex vivo* studies [32–38]. Model predictions can then be evaluated by comparing with observed data from patients on ECLS and the drug of interest.

## Conclusion

Milrinone is rapidly cleared by CRRT circuits and may require altered dosing in critically ill patients being supported by this therapy. Clinical studies that incorporate patient pathophysiology are needed to inform optimal drug dosing. By contrast, milrinone is not measurably adsorbed to components of an ECMO circuit, thus dosing adjustments to account for adsorption to the ECMO circuit are likely unnecessary. These results will inform clinical studies of optimal dosing in patients requiring ECMO and CRRT and improve the safety and efficacy of milrinone in these vulnerable populations.

## Data Availability

The research data associated with this article are included in the article.

## Supplementary material

The supplementary material of this article is available at https://ject.edpsciences.org/10.1051/ject/2024014/olm. *Supplementary Table 1*: Milrinone control plasma concentration (ng/mL). *Supplementary Table 2*: Milrinone ECMO plasma concentration (ng/mL). *Supplementary Table 3*: Milrinone CRRT plasma concentrations (ng/mL).

## References

[1] Hayes LW, Oster RA, Tofil NM, Tolwani AJ. Outcomes of critically ill children requiring continuous renal replacement therapy. J Crit Care. 2009;24(3):394–400. 10.1016/j.jcrc.2008.12.017. 19327959

[2] Tandukar S, Palevsky PM. Continuous renal replacement therapy: who, when, why, and how. Chest. 2019;155(3):626–638. 10.1016/j.chest.2018.09.004. 30266628 PMC6435902

[3] Hoffman TM, Wernovsky G, Atz AM, et al. Efficacy and safety of milrinone in preventing low cardiac output syndrome in infants and children after corrective surgery for congenital heart disease. Circulation. 2003;107(7):996–1002. 10.1161/01.CIR.0000051365.81920.28. 12600913

[4] Vogt W, Läer S. Prevention for pediatric low cardiac output syndrome: results from the European survey EuLoCOS-Paed. Pediatr Anesth. 2011;21(12):1176–1184. 10.1111/j.1460-9592.2011.03683.x. 21851475

[5] Hornik CP, Yogev R, Mourani PM, et al. Population pharmacokinetics of milrinone in infants, children, and adolescents. J Clin Pharmacol. 2019;59(12):1606–1619. 10.1002/jcph.1499. 31317556 PMC6813877

[6] Fredholm M, Jörgensen K, Houltz E, Ricksten SE. Inotropic and lusitropic effects of levosimendan and milrinone assessed by strain echocardiography – a randomised trial. Acta Anaesthesiol Scand. 2018;62(9):1246–1254. 10.1111/aas.13170. 29926912

[7] Yano M, Kohno M, Ohkusa T, et al. Effect of milrinone on left ventricular relaxation and Ca^2+^ uptake function of cardiac sarcoplasmic reticulum. Am J Physiol Heart Circ Physiol. 2000;279(4):H1898–H1905. 10.1152/ajpheart.2000.279.4.H1898. 11009478

[8] Honerjäger P, Nawrath H. Pharmacology of bipyridine phosphodiesterase III inhibitors. Eur J Anaesthesiol Suppl. 1992;5:7–14. 1600969

[9] Kim M, Seong SW, Song PS, et al. Inodilators may improve the in-hospital mortality of patients with cardiogenic shock undergoing veno-arterial extracorporeal membrane oxygenation. J Clin Med. 2022;11(17):4958. 10.3390/jcm11174958. 36078888 PMC9456701

[10] Rodenas-Alesina E, Luis Scolari F, Wang VN, et al. Improved mortality and haemodynamics with milrinone in cardiogenic shock due to acute decompensated heart failure. ESC Heart Fail. 2023;10(4):2577–2587. 10.1002/ehf2.14379. 37322827 PMC10375068

[11] Ricci Z, Goldstein SL. Pediatric continuous renal replacement therapy. Contrib Nephrol. 2016;187:121–130. 10.1159/000442370. 26881430

[12] Watson RS, Crow SS, Hartman ME, Lacroix J, Odetola FO. Epidemiology and outcomes of pediatric multiple organ dysfunction syndrome. Pediatr Crit Care Med. 2017;18(3_suppl Suppl 1):4–16. 10.1097/PCC.0000000000001047. PMC5334773 28248829

[13] Nasr VG, Raman L, Barbaro RP, et al. Highlights from the Extracorporeal Life Support Organization Registry: 2006–2017. ASAIO J. 2019;65(6):537–544. 10.1097/MAT.0000000000000863. 30074497

[14] Raffaeli G, Pokorna P, Allegaert K, et al. Drug disposition and pharmacotherapy in neonatal ECMO: from fragmented data to integrated knowledge. Front Pediatr. 2019;7:360. 10.3389/fped.2019.00360. 31552205 PMC6733981

[15] Nolin TD, Aronoff GR, Fissell WH, et al. Pharmacokinetic assessment in patients receiving continuous RRT: perspectives from the kidney health initiative. Clin J Am Soc Nephrol. 2015;10(1):159–164. 10.2215/CJN.05630614. 25189923 PMC4284416

[16] Hunt JP, McKnite AM, Green DJ, Whelan AJ, Imburgia CE, Watt KM. Interaction of ceftazidime and clindamycin with extracorporeal life support. J Infect Chemother. 2023;29(12):1119–1125. 10.1016/j.jiac.2023.08.007. 37572979 PMC11160944

[17] Burkhardt BE, Rücker G, Stiller B. Prophylactic milrinone for the prevention of low cardiac output syndrome and mortality in children undergoing surgery for congenital heart disease. Cochrane Database Syst Rev. 2015;3:CD00951. 10.1002/14651858.CD009515.pub2. PMC11032183 25806562

[18] Bishara T, Seto WTW, Trope A, Parshuram CS. Use of milrinone in critically ill children. Can J Hosp Pharm. 2010;63(6):420–428. 10.4212/cjhp.v63i6.960. 22479014 PMC3004699

[19] DrugBank Version 45.1.1. Drug and drug target database. http://www.drugbank.ca. Accessed 26 Aug 2023.

[20] Cone J, Wang S, Tandon N, et al. Comparison of the effects of cilostazol and milrinone on intracellular cAMP levels and cellular function in platelets and cardiac cells. J Cardiovasc Pharmacol. 1999;34(4):497–504. 10.1097/00005344-199910000-00004. 10511123

[21] Kuthe A, Magert H, Uckert S, Forssmann WG, Stief CG, Jonas U. Gene expression of the phosphodiesterases 3A and 5A in human corpus cavernosum penis. Eur Urol. 2000;38(1):108–114. 10.1159/000020262. 10859452

[22] Baruch L, Patacsil P, Hameed A, Pina I, Loh E. Pharmacodynamic effects of milrinone with and without a bolus loading infusion. Am Heart J. 2001;141(2):14A–21A. 10.1067/mhj.2001.111404. 11174341

[23] Bailey JM, Levy JH, Kikura M, Szlam F, Hug CC. Pharmacokinetics of intravenous milrinone in patients undergoing cardiac surgery. Anesthesiology. 1994;81(3):616–622. 10.1097/00000542-199409000-00014. 8092507

[24] Cox ZL, Calcutt MW, Morrison TB, Akers WS, Davis MB, Lenihan DJ. Elevation of plasma milrinone concentrations in stage D heart failure associated with renal dysfunction. J Cardiovasc Pharmacol Ther. 2013;18(5):433–438. 10.1177/1074248413489773. 23695773

[25] Lemaitre F, Hasni N, Leprince P, et al. Propofol, midazolam, vancomycin and cyclosporine therapeutic drug monitoring in extracorporeal membrane oxygenation circuits primed with whole human blood. Crit Care. 2015;19:40. 10.1186/s13054-015-0772-5. 25886890 PMC4335544

[26] Shekar K, Roberts JA, Mcdonald CI, et al. Protein-bound drugs are prone to sequestration in the extracorporeal membrane oxygenation circuit: results from an ex vivo study. Crit Care. 2015;19(1):164. 10.1186/s13054-015-0891-z. 25888449 PMC4407324

[27] Mehta NM, Halwick DR, Dodson BL, Thompson JE, Arnold JH. Potential drug sequestration during extracorporeal membrane oxygenation: results from an ex vivo experiment. Intensive Care Med. 2007;33(6):1018–1024. 10.1007/s00134-007-0606-2. 17404709

[28] Dallefeld SH, Sherwin J, Zimmerman KO, Watt KM. Dexmedetomidine extraction by the extracorporeal membrane oxygenation circuit: results from an in vitro study. Perfusion. 2020;35(3):209–216. 10.1177/0267659119868062. 31431126 PMC7275646

[29] Preston TJ, Ratliff TM, Gomez D, et al. Modified surface coatings and their effect on drug adsorption within the extracorporeal life support circuit. J Extra Corpor Technol. 2010;42(3):199–202. 21114222 PMC4679959

[30] Green DJ, Watt KM, Fish DN, McKnite A, Kelley W, Bensimhon AR. Cefepime extraction by extracorporeal life support circuits. J Extra Corpor Technol. 2022;54(3):212–222. 10.1182/ject-212-222. 36742220 PMC9891479

[31] Imburgia CE, Rower JE, Green DJ, et al. Remdesivir and GS-441524 extraction by ex vivo extracorporeal life support circuits. ASAIO J. 2022;68(9):1204–1210. 10.1097/MAT.0000000000001616. 34799526 PMC9110562

[32] Onichimowski D, Nosek K, Ziółkowski H, Jaroszewski J, Pawlos A, Czuczwar M. Adsorption of vancomycin, gentamycin, ciprofloxacin and tygecycline on the filters in continuous renal replacement therapy circuits: in full blood in vitro study. J Artif Organs. 2021;24(1):65–73. 10.1007/s10047-020-01214-8. 33033945 PMC7889537

[33] Onichimowski D, Ziółkowski H, Nosek K, Jaroszewski J, Rypulak E, Czuczwar M. Comparison of adsorption of selected antibiotics on the filters in continuous renal replacement therapy circuits: in vitro studies. J Artif Organs. 2020;23(2):163–170. 10.1007/s10047-019-01139-x. 31630269 PMC7228979

[34] Kumar A, Mann HJ, Keshtgarpour M, et al. In vitro characterization of oritavancin clearance from human blood by low-flux, high-flux, and continuous renal replacement therapy dialyzers. Int J Artif Organs. 2011;34(11):1067–1074. 10.5301/ijao.5000008. 22183520

[35] Baud FJ, Houzé P, Raphalen JH, et al. Diafiltration flowrate is a determinant of the extent of adsorption of amikacin in renal replacement therapy using the ST150^®^-AN69 filter: An in vitro study. Int J Artif Organs. 2020;43(12):758–766. 10.1177/0391398820911928. 32356511

[36] Choi G, Gomersall CD, Lipman J, et al. The effect of adsorption, filter material and point of dilution on antibiotic elimination by haemofiltration an in vitro study of levofloxacin. Int J Antimicrob Agents. 2004;24(5):468–472. 10.1016/j.ijantimicag.2004.06.005. 15519479

[37] Wildschut ED, Ahsman MJ, Allegaert K, Mathot RAA, Tibboel D. Determinants of drug absorption in different ECMO circuits. Intensive Care Med. 2010;36(12):2109–2116. 10.1007/s00134-010-2041-z. 20862453 PMC2981740

[38] Gist KM, Mizuno T, Goldstein SL, Vinks A. Retrospective evaluation of milrinone pharmacokinetics in children with kidney injury. Ther Drug Monit. 2015;37(6):792–796. 10.1097/FTD.0000000000000214. 25860636

[39] O’Hanlon CJ, Sumpter A, Anderson BJ, Hannam JA. Time-varying clearance in milrinone pharmacokinetics from premature neonates to adolescents. Clin Pharmacokinet. 2024;63:695–706. 10.1007/s40262-024-01372-5. 38613610 PMC11106138

[40] Taniguchi T, Shibata K, Saito S, Matsumoto H, Okeie K. Pharmacokinetics of milrinone in patients with congestive heart failure during continuous venovenous hemofiltration. Intensive Care Med. 2000;26(8):1089–1093. 10.1007/s001340051322. 11030165

[41] Watt KM, Cohen-Wolkowiez M, Barrett JS, et al. Physiologically based pharmacokinetic approach to determine dosing on extracorporeal life support: fluconazole in children on ECMO. CPT Pharmacometrics Syst Pharmacol. 2018;7(10):629–637. 10.1002/psp4.12338. 30033691 PMC6202466

