# Supplementary material for: Interaction of milrinone with extracorporeal life support
Source: J Extra Corpor Technol. 2024 Dec 20;56(4):167–73. doi: 10.1051/ject/2024014 (PMC11661780; doi:10.1051/ject/2024014)
Supplement: Supplementary file 1 — Supplementary Table 1: Milrinone control plasma concentration (ng/mL). Supplementary Table 2: Milrinone ECMO plasma concentration (ng/mL). Supplementary Table 3: Milrinone CRRT plasma concentrations (ng/mL). [file ject-56-167-s1.pdf]

Interaction of Milrinone with Extracorporeal Life Support

Aviva J. Whelan[1,2], Sabiha Mim[3], J. Porter Hunt[1], Autumn M. McKnite[4], Danielle J. Green[1,2], Carina E. Imburgia[1], Jeremiah D. Momper[5], Gideon Stitt[1], Kevin M. Watt[1,2]

[1]Division of Clinical Pharmacology, Department of Pediatrics, University of Utah, Salt Lake City, Utah, USA
[2] Division of Pediatric Critical Care, Department of Pediatrics, University of Utah, Salt Lake City, Utah, USA
[3] Pharmacometric Research Group, Department of Pharmacy, Uppsala University, Uppsala, Sweden
[4] Department of Pharmacology and Toxicology, University of Utah, Salt Lake City, Utah, USA
[5] Skaggs School of Pharmacy and Pharmaceutical Sciences, University of California San Diego, La Jolla, CA

*** Correspondence:**
Kevin M. Watt
kevin.watt@hsc.utah.edu

EXPERIMENTAL RESULTS

Supplementary Table 1: Milrinone Control Plasma Concentration (ng/mL)

Time	Standard Control		
	Run 1	Run 2	Run 3
1 min	188.1	178.3	192.7
5 min	186.7	188.5	188.9
15 min	188.2	188.9	186.8
30 min	195.0	171.9	194.5
1 hr	193.9	163.2	194.8
2 hr	179.8	179.9	195.2
3 hr	196.2	174.8	177
4 hr	198.1	208.0	209.4
5 hr	190.7	201.6	179.7
6 hr	198.8	186.6	187.9

Supplementary Table 2: Milrinone ECMO Plasma Concentration (ng/mL)

Time	ECMO Circuit		
	Run 1	Run 2	Run 3
1 min	122.6	113.9	135.1
5 min	114.8	129	130.5
15 min	109	110.9	126.4
30 min	125.5	116.3	124.3
1 hr	108.5	116.4	141.8
2 hr	128.6	134.2	134.2
3 hr	119.5	130	122.9
4 hr	128.8	149.3	140.1
5 hr	106.1	125.4	127.9
6 hr	126	123.5	119.9

Note - All samples below the limit of quantification were assigned a value of 0.0

Supplementary Table 3: Milrinone CRRT Plasma Concentrations (ng/mL)

Time	CRRT Circuit – Plasma		
	Run 1	Run 2	Run 3
1 min	316.8	255.5	256.8
5 min	97.5	100.9	86.6
15 min	69.7	73.0	62.5
30 min	44.6	51.1	34.6
1 hr	21.3	21.2	12.2
2 hr	4.5	3.6	2.6
3 hr	0.0	0.0	0.0
4 hr	0.0	0.0	0.0
5 hr	0.0	0.0	0.0
6 hr	0.0	0.0	0.0

Note - All samples below the limit of quantification were assigned a value of 0.0

Supplementary Table 4: Milrinone CRRT Effluent Concentrations (ng/mL)

Time	CRRT Circuit – Effluent		
	Run 1	Run 2	Run 3
1 min	188.7	333.8	306.5
5 min	68.8	66.9	63.9
15 min	43.9	49.3	52
30 min	29.4	32.7	27.2
1 hr	7.3	13.2	8.6
2 hr	0.0	1.6	0.0
3 hr	0.0	0.0	0.0
4 hr	0.0	1.3	0.0
5 hr	0.0	0.0	0.0
6 hr	0.0	0.0	0.0

Note - All samples below the limit of quantification were assigned a value of 0.0
